# Prevalence and clinical impact of disseminated intravascular coagulation in acute aortic dissection: a nationwide cohort study

**DOI:** 10.1016/j.rpth.2024.102656

**Published:** 2024-12-16

**Authors:** Shuhei Murao, Yutaka Umemura, Hirotaka Mori, Yoshinobu Seki, Takayuki Ikezoe, Kohji Okamoto, Satoshi Fujimi, Kazuma Yamakawa

**Affiliations:** 1 Department of Emergency and Critical Care, Osaka General Medical Center, Osaka, Japan; 2 Department of Biostatistics, Graduate School of Medicine, Hokkaido University, Sapporo, Japan; 3 Department of Hematology, Uonuma Institute of Community Medicine, Niigata University Medical and Dental Hospital, Niigata, Japan; 4 Department of Surgery, Kitakyushu City Yahata Hospital, Fukuoka, Japan; 5 Department of Emergency and Critical Care Medicine, Osaka Medical and Pharmaceutical University, Osaka, Japan

**Keywords:** aortic dissection, coagulopathy, disseminated intravascular coagulation (DIC), ISTH DIC, JAAM-2 DIC

## Abstract

**Background:**

Acute aortic dissection is a life-threatening cardiovascular emergency with high mortality rates. Disseminated intravascular coagulation (DIC) is a critical complication in patients with acute aortic dissection; however, its incidence and impact on outcomes remain inconclusive.

**Objectives:**

This study aimed to evaluate DIC prevalence and prognosis in patients with aortic dissection.

**Methods:**

We conducted a multicenter retrospective cohort study using data from the Japan Medical Data Center claims database between 2014 and 2022. DIC was diagnosed based on the criteria of the Japanese Association for Acute Medicine (JAAM-2) and the International Society on Thrombosis and Haemostasis (ISTH). We compared the in-hospital mortality between patients with and without DIC and assessed the impact of coagulopathy using various coagulation profiles.

**Results:**

Among the 3037 patients, 40% underwent surgery and 60% did not undergo surgery. The prevalence rates of JAAM-2 DIC and ISTH DIC were 21% and 9.4%, respectively. In-hospital mortality was significantly higher in the DIC group than in the non-DIC group, and this trend was consistently observed in the surgery and nonsurgery groups. Increased DIC scores correlated with higher in-hospital mortality. With the progression of coagulopathy, characterized by thrombocytopenia, elevated prothrombin time-international normalized ratio, prolonged activated partial thromboplastin time, increased D-dimer, and decreased fibrinogen levels, in-hospital mortality also increased.

**Conclusion:**

The presence of DIC, as identified by both the JAAM-2 and ISTH criteria, was associated with increased in-hospital mortality in patients with acute aortic dissection. Therefore, further studies are needed to improve the clinical outcomes of these patients.

## Essentials

• Epidemiology of disseminated intravascular coagulation (DIC) in aortic dissection is unknown.
• This was a multicenter retrospective cohort study conducted in Japan.
• The presence of DIC was associated with increased in-hospital mortality.
• DIC increased mortality regardless of surgery, nonsurgery, or type of surgery.

## 1 Introduction

Acute aortic dissection is an urgent cardiovascular condition characterized by high morbidity and mortality rates, often accompanied by excessive bleeding complications [1,2]. Contact between blood and the false lumen of the dissected aorta can lead to dysregulation of the coagulation system, resulting in disseminated intravascular coagulation (DIC) [3,4]. The prevalence of DIC varies markedly depending on the underlying condition, with reported rates of approximately 30% to 60% in patients with sepsis, 30% to 40% in those with head trauma, and 10% in patients with cancer [[5], [6], [7]].

In previous studies, some patients with acute type A dissection exhibited significant reduction in platelets and coagulation factors at initial presentation and postoperatively, and low preoperative fibrinogen levels have been correlated with increased risk of neurologic complications [[8], [9], [10]]. Recent cohort studies demonstrated that increased DIC scores were associated with a larger diameter and length of the false lumen, increased thickness of the dissection membrane, and communicating type A aortic dissection [4,11]. Nevertheless, the incidence and overall clinical impact of DIC on survival outcomes remain uncertain based on the studies involved.

Furthermore, the current guidelines on aortic aneurysm and dissection refer to the difficulty of hemorrhagic control in patients with DIC with enhanced fibrinolysis; however, no clear diagnosis or treatment guidance for DIC has been provided, owing to limited evidence [12]. Thus, the present study aimed to investigate the prevalence and prognosis of DIC in patients with acute aortic dissection and to characterize these conditions based on their coagulation profiles using a nationwide database in Japan.

## 2 Methods

### 2.1 Data source

We conducted a retrospective cohort study using data from the Japan Medical Data Center claims database (Tokyo, Japan) between 2014 and 2022 [13,14]. This database is a significant repository of health insurance claims developed in collaboration with >60 insurance providers, with a cumulative population of approximately 17 million. The database contains integrated medical and pharmacy claims data, including demographics, diagnoses, examinations, drug prescriptions, and laboratory data. The diagnoses for insurance claims were coded according to the International Statistical Classification of Diseases and Related Health Problems, 10th Revision.

### 2.2 Study population

We identified patients with acute aortic dissection (International Statistical Classification of Diseases and Related Health Problems, 10th Revision code, I71.0) recorded as either the “main diagnosis,” “admission-precipitating diagnosis,” or “most resource-consuming diagnosis.” Patients with planned admissions or missing age or survival outcome data were excluded. We also excluded patients for whom data on the Japanese Association for Acute Medicine (JAAM-2) DIC score, including platelet count, prothrombin time-international normalized ratio (PT-INR), fibrin degradation products (FDPs), or D-dimer measured on admission day, were missing.

### 2.3 Data collection

We collected demographic data (age, sex, and body mass index); comorbidities (myocardial infarction, heart failure, chronic lung disease, cerebrovascular disease, peripheral vascular disease, diabetes mellitus, and chronic kidney disease); surgical information (open or graft surgery, surgery for type A or type B dissection); coagulation tests (platelet counts, prothrombin time, activated partial thromboplastin time [aPTT], fibrinogen, FDPs, D-dimer, antithrombin, thrombin antithrombin III complex, and plasmin-α2 plasmin inhibitor complex); Sequential Organ Failure Assessment score, JAAM-2 DIC score, and the International Society on Thrombosis and Haemostasis (ISTH) DIC score; intensive care unit (ICU) admission, vasopressor use, mechanical ventilation and renal replacement therapy, transfusion information (red blood cell, fresh frozen plasma, and platelets). The outcome data included in-hospital mortality, length of ICU stay, and length of hospital stay.

DIC scores were determined upon admission using 2 established scoring systems, the JAAM-2 and ISTH DIC criteria, as shown in Table 1 [15,16]. In the JAAM-2 DIC criteria recently proposed by JAAM, the systemic inflammatory response syndrome score component was omitted from the original version, and the cutoff value for diagnosing DIC was set at ≥3 points [15]. DIC was diagnosed based on the ISTH DIC score when patients scored ≥5 points. As a subanalysis, we also calculated the modified ISTH (m-ISTH) score by excluding fibrinogen values from the original ISTH score. A score of ≥4 points was considered positive. In the ISTH criteria, the FDP thresholds defined in the JAAM-2 criteria were adopted as markers of fibrin involvement: FDP levels <10, 10 to <25, and ≥25 μg/mL corresponded to no, moderate, and strong increases in fibrin-related markers, respectively.

**Table 1:** JAAM-2 DIC and ISTH DIC scoring systems.

Parameter	Points	JAAM-2 DIC	ISTH DIC
Platelet count	3	<80 × 10^3^/μL or >50% decrease/24 h	–
	2	–	<50 × 10^3^/μL
	1	≥80, <120 × 10^3^/μL or 30%-50% decrease/24 h	≥50, <100 × 10^3^/μL
FDP or D-dimer	3	FDP ≥25 μg/mL or D-dimer ≥15 μg/mL	Strong increase
	2	–	Moderate increase
	1	FDP ≥10, <25 μg/mL or D-dimer ≥6, <15 μg/mL	–
Prothrombin time	2	–	Prolonged PT ≥6 s
	1	PT-INR ≥1.2	Prolonged PT ≥3, <6 s
Fibrinogen	1	–	<100 mg/dL
DIC criteria positive		3 points	5 points

DIC, disseminated intravascular coagulation; FDP, fibrin degradation product; ISTH, International Society on Thrombosis and Haemostasis; JAAM-2, Japanese Association for Acute Medicine; PT, prothrombin time; PT-INR, prothrombin time-international normalized ratio.

### 2.4 Statistical analysis

The baseline variables were compared between patients with and without DIC. The analysis was conducted for each cohort for which the JAAM-2 and ISTH scores could be calculated. The cohorts were separated based on the availability of specific data required to apply the JAAM-2 and ISTH DIC criteria. Quantitative parameters are reported as medians and interquartile ranges (25^th^-75th percentiles) and compared using the Mann–Whitney U-test. Qualitative parameters are expressed as numbers and percentages and were compared using the chi-squared test or Fisher’s exact test, as appropriate. Clinical outcomes, including in-hospital mortality, hospital length of stay, and ICU length of stay, were evaluated in patients with and without DIC and stratified according to surgical information. The positive and mortality rates for the JAAM-2, ISTH, and m-ISTH DIC scores were calculated. We also evaluated the number of patients for each DIC subscore and its association with in-hospital mortality. In-hospital mortality rates were also evaluated for each score on the JAAM-2 and ISTH DIC scales. Logistic regression analysis was performed to assess the association between the severity of each coagulopathy in individual coagulation profiles and in-hospital mortality. The trend in hemostatic status between survivors and nonsurvivors was compared from days 1 to 4.

## 3 Results

**Figure 1:**
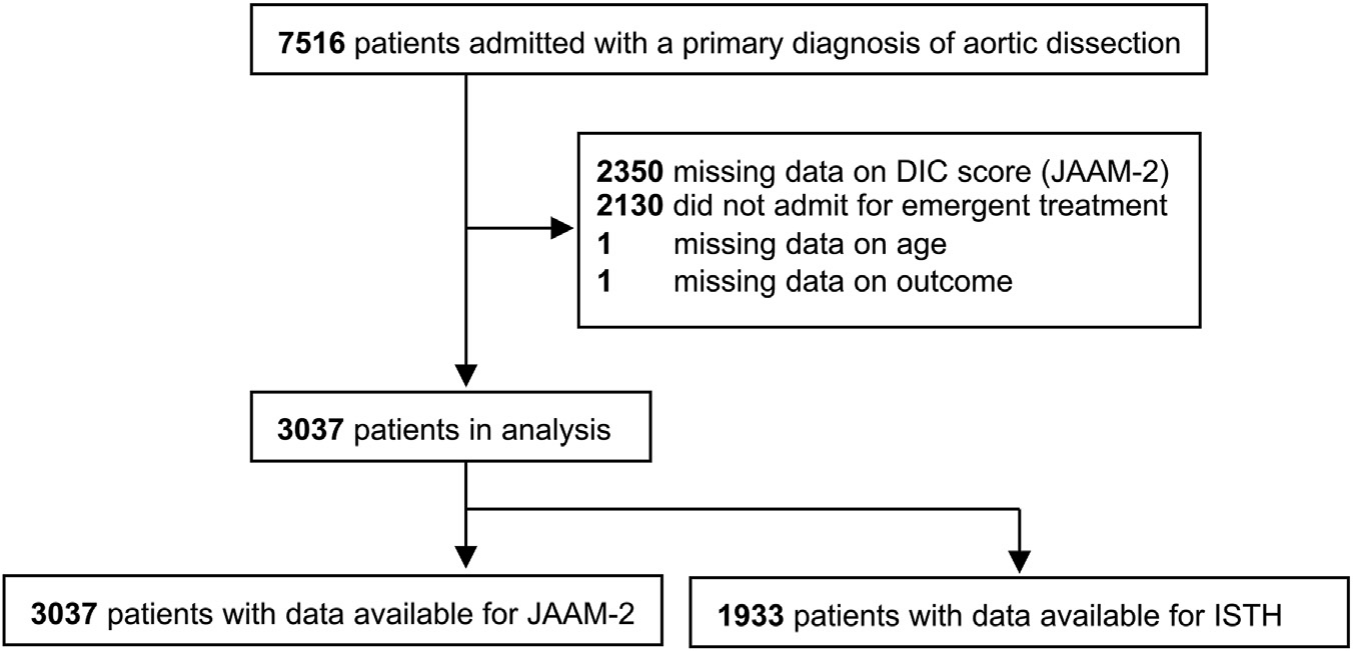
Patient flow chart. DIC, disseminated intravascular coagulation; ISTH, International Society on Thrombosis and Haemostasis; JAAM-2, Japanese Association for Acute Medicine.

A total of 7516 patients with aortic dissection were enrolled in the present study. We excluded 2130 patients who were not admitted for emergency treatment and 2350 patients with data missing to calculate the JAAM-2 DIC score. A total of 3037 patients were included in the analysis, and data for calculating the ISTH DIC score were available for 1933 patients (Figure 1). Table 2 lists the demographic and clinical characteristics of the patients. The median age of the patients was 73 years (interquartile range, 62-81 years), and 56% were men. Among the patients, 40% received surgery whereas 60% did not receive surgery. Among all patients, 32% underwent open surgery, and 35% underwent surgery specifically for type A aortic dissection.

**Table 2:** Demographic and clinical characteristics of the study patients.

Characteristic	All patients	JAAM-2			ISTH		
		DIC	non-DIC	*P*	DIC	non-DIC	*P*
Number of patients (%)	3037	637 (21)	2400 (79)		182 (9.4)	1751 (91)	
Age, y	73 (62-81)	75 (66-83)	72 (61-81)	<.001	73 (65-82)	72 (60-80)	.08
Sex, male	1705 (56)	1390 (57)	315 (49)	<.001	92 (51)	969 (55)	.25
BMI, kg/m^2^	23 (20.6-25.6)	22.9 (20.4-25.5)	23.2 (20.7-25.7)	.08	23.1 (20.6-25.4)	23.1 (20.5-25.8)	.74
Comorbidity							
Myocardial infarction	124 (4.8)	19 (3.0)	105 (4.4)	.11	6 (3.3)	78 (4.5)	.47
Heart failure	1086 (36)	204 (32)	882 (37)	.03	62 (34)	627 (36)	.64
Chronic lung diseases	308 (10)	53 (8.3)	255 (11)	.09	7 (3.9)	167 (9.5)	.01
Cerebral vascular diseases	549 (18)	142 (22)	407 (17)	.002	58 (32)	298 (17)	<.001
Diabetes mellitus	366 (12)	66 (10)	300 (13)	.14	21 (12)	211 (12)	.84
Chronic kidney diseases	248 (8.2)	52 (8.2)	196 (8.2)	1	12 (7.8)	139 (7.9)	.52
Surgery, *n* (%)	1217 (40)	364 (30)	853 (70)		142 (14)	867 (86)	
Open surgery	955 (32)	297 (31)	658 (69)		132 (16)	678 (84)	
Endovascular surgery	295 (9.7)	85 (29)	210 (71)		18 (8.0)	207 (92)	
Open and endovascular surgery	33 (1.1)	18 (55)	15 (45)		8 (31)	18 (69)	
Surgery for type A	1066 (35)	347 (33)	719 (67)		138 (15)	775 (85)	
Surgery for type B	151 (5.0)	17 (11)	134 (89)		4 (4.1)	92 (96)	
Conservative treatment	1820 (60)	273 (15)	1547 (85)		40 (4.3)	884 (96)	
Coagulation test on admission							
Platelet count, 10^3^/μL (n=3037)	17.7 (13.2-22.2)	10.3 (7.5-13.4)	19.0 (15.4-23.3)	<.001	8.0 (5.5-11.5)	17.7 (13.5-21.9)	<.001
PT, s (*n* = 2890)	12.4 (11.6-13.8)	14.4 (12.7-16.5)	12.2 (11.5-13.2)	<.001	17.3 (15.4-20.7)	12.5 (11.7-13.6)	<.001
PT-INR (*n* = 1943)	1.06 (0.97-1.19)	1.28 (1.15-1.47)	1.03 (0.96-1.11)	<.001	1.36 (1.19-1.68)	1.06 (0.97-1.17)	<.001
APTT, s (*n* = 2728)	29.7 (26.7-32.6)	31.1 (28.7-39.6)	29.0 (26.2-31.4)	<.001	32.7 (30.0-40.8)	29.7 (26.7-32.2)	<.001
Fibrinogen, mg/dL (*n* = 1933)	279 (201-370)	185 (124-261)	304 (236-402)	<.001	112 (82-186)	290 (220-382)	<.001
FDP, μg/mL (*n* = 1538)	23.3 (9.2-71.4)	78.3 (40.5-232.3)	14.8 (7.2-38.6)	<.001	143.5 (60.5-467.2)	21.0 (9.0-58.9)	<.001
D-dimer, μg/mL (*n* = 2860)	8.6 (3.1-24.8)	36.5 (19.4-85.6)	6.0 (2.6-13.6)	<.001	52.0 (24.8-155)	8.1 (3.1-21.5)	<.001
FDP/D-dimer ratio (*n* = 1432)	2.6 (2.2-3.2)	2.6 (2.2-3.3)	2.6 (2.1-3.2)	.31	2.6 (2.2-3.4)	2.5 (2.2-3.2)	.96
Antithrombin, % (*n* = 626)	88 (79-97)	83 (71-93)	89 (82-99)	<.001	88 (80-98)	73 (58-81)	<.001
TAT, ng/mL (*n* = 180)	26.2 (12.2-50.3)	35.2 (22.3-74.9)	25.5 (10.6-48.0)	.007	51.5 (28.0-74.9)	25.5 (11.5-50.0)	.32
PIC, μg/mL (*n* = 1)	0.5 (0.5-0.5)	NA	NA		NA	NA	
SOFA score (*n* = 658)	7 (2-10)	9 (5-11)	6 (2-10)	<.001	11 (5-16)	8 (3-11)	<.001
JAAM-2 DIC score (*n* = 3037)	2 (0-3)	4 (4-5)	1 (0-3)	<.001	5 (4-7)	2 (0-3)	<.001
ISTH DIC score (*n* = 1933)	3 (0-3)	4 (3-5)	2 (0-3)	<.001	5 (5-6)	2 (0-3)	<.001
Heparin, *n* (%)	1633 (54)	399 (63)	1234 (51)	<.001	146 (80)	1066 (61)	<.001
Antithrombin, *n* (%)	36 (1.2)	13 (2.0)	23 (1.0)	.03	5 (2.8)	24 (1.4)	.15
ICU admission, *n* (%)	1672 (55)	393 (62)	1279 (53)	<.001	140 (77)	1079 (62)	<.001
Vasopressor use, *n* (%)	1155 (38)	390 (61)	765 (32)	<.001	137 (75)	725 (41)	<.001
Mechanical ventilation, *n* (%)	1265 (42)	411 (65)	854 (36)	<.001	143 (79)	839 (48)	<.001
Renal replacement therapy	180 (5.9)	72 (11)	108 (4.5)	<.001	37 (20)	109 (6.2)	<.001
Red blood cell, U	10 (6-16)	12 (8-18)	10 (6-14)	<.001	14 (10-22)	10 (6-14)	<.001
Fresh frozen plasma, U	10 (8-16)	12 (10-20)	10 (8-16)	<.001	10 (8-20)	10 (8-16)	.02

APTT, activated partial thromboplastin time; BMI, body mass index; DIC, disseminated intravascular coagulation; FDP, fibrin degradation product; ICU, intensive care unit; ISTH, International Society on Thrombosis and Haemostasis; JAAM-2, Japanese Association for Acute Medicine; PIC, plasmin-α2 plasmin inhibitor complex; PT, prothrombin time; PT-INR, prothrombin time-international normalized ratio; SOFA, Sequential Organ Failure Assessment; TAT, thrombin antithrombin III complex.

In the overall patient cohort, the prevalence of JAAM-2 DIC and ISTH DIC was 21% (637/3037 patients) and 9.4% (182/1933 patients), respectively. Of the patients who underwent surgery, 30% were diagnosed with JAAM-2 DIC and 14% with ISTH DIC. Among those who did not undergo surgery, 15% were diagnosed with JAAM-2 DIC and 4.3% with ISTH DIC. For both criteria, patients in the DIC group showed significant alterations in their coagulation profiles, including decreased platelet counts and antithrombin levels and increased PT-INR, aPTT, FDPs, and D-dimer, when compared with those in the non-DIC group. There was a higher rate of heparin administration in the DIC group than in the non-DIC group. Patients in the DIC group more frequently required ICU admission, vasopressor support, mechanical ventilation, and renal replacement therapy and required higher volumes of transfusion.

**Figure 2:**
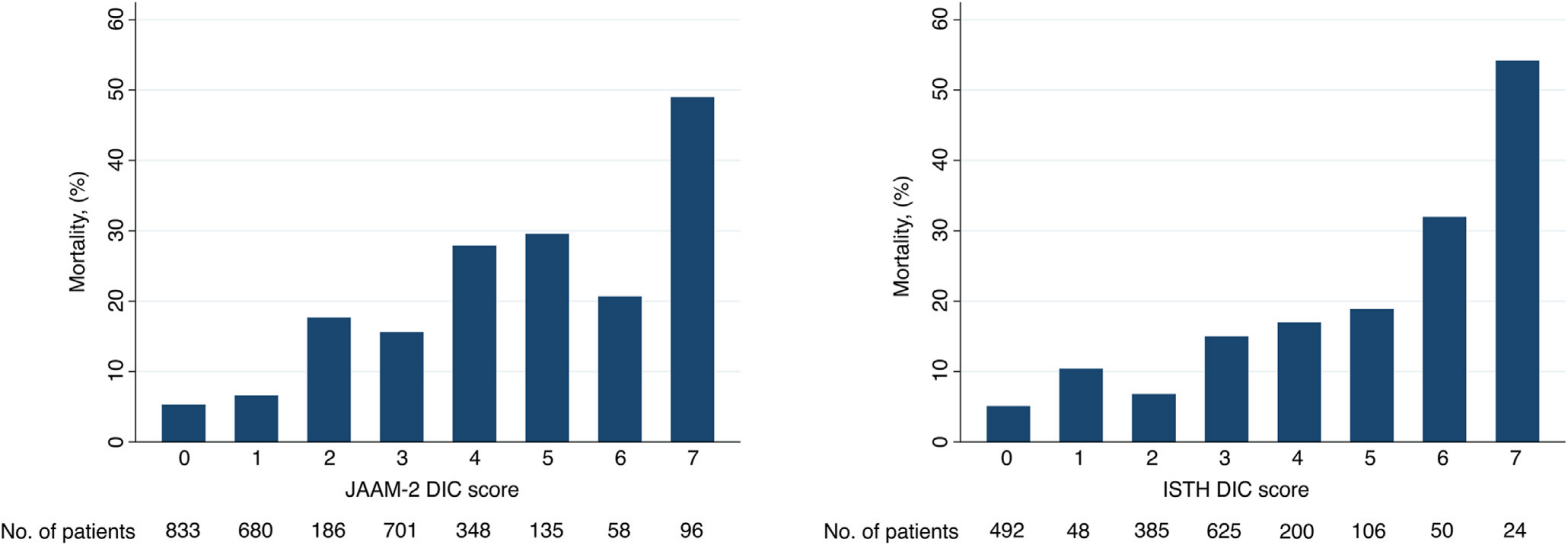
Mortality by JAAM-2 DIC score and ISTH DIC score among patients with acute aortic dissection. DIC, disseminated intravascular coagulation; ISTH, International Society on Thrombosis and Haemostasis; JAAM-2, Japanese Association for Acute Medicine.

Table 3 shows the clinical outcomes of the patients with and without DIC. For both criteria, patients with DIC showed significantly higher in-hospital mortality rates than those without non-DIC (JAAM-2, 31% vs 9.6%, *P* < .001; ISTH, 27% vs 11%, *P* < .001). Increased in-hospital mortality was also observed in patients who underwent surgery (JAAM-2, 10% vs 5.6%, *P* = .003; ISTH, 16% vs 5.3%, *P* < .001). Stratified analyses by the type of surgical intervention revealed consistently higher mortality rates within the DIC group across procedures, such as open surgery, graft surgery, and surgery, specifically for type A aortic dissection. Table 4 shows fibrinogen levels were below the threshold in 5.4% of cases using the ISTH score. After excluding fibrinogen, the m-ISTH DIC score was positive in 16% of patients, with a corresponding mortality rate of 32%. As shown in Figure 2, the in-hospital mortality rates increased with an increase in both the JAAM-2 DIC and ISTH DIC scores.

**Table 3:** Clinical outcome of patients with and without DIC stratified by surgical status.

Outcome	All patients	JAAM-2			ISTH		
		DIC	non-DIC	*P*	DIC	non-DIC	*P*
Overall, *n* (%)	3037	637 (21)	2400 (79)		182 (9.4)	1751 (91)	
In-hospital mortality, %	14	31	9.6	<.001	27	11	<.001
Length of ICU stay, d, mean (CI)	5 (3-8)	5 (3-9)	5 (3-7)	<.001	5 (4-8)	5 (3-8)	.11
Length of hospital stay, days, mean (CI)	18 (12-27)	20 (4-32)	18 (13-26)	.64	25 (14-36)	19 (13-28)	<.001
Surgery, *n* (%)	1217 (40.1)	364 (30)	853 (70.1)		142 (14)	867 (86)	
In-hospital mortality, %	7.1	10	5.6	.003	16	5.3	<.001
Length of ICU stay, d, mean (CI)	5 (4-9)	6 (4-9)	5 (3-9)	.21	5 (4-8)	5 (3-8)	1
Length of hospital stay, d, mean (CI)	25 (17-37)	26 (18-42)	23 (17-35)	.002	26 (18-36)	23 (17-35)	.11
Open surgery, *n* (%)	955 (31.5)	297 (31)	658 (69)		132 (16)	678 (84)	
In-hospital mortality, %	6.8	9.4	5.6	.03	14	4.9	<.001
Length of ICU stay, d, mean (CI)	5 (3-8)	6 (4-9)	5 (3-8)	.06	5 (4-8)	5 (3-8)	.82
Length of hospital stay, d, mean (CI)	25 (18-37)	26 (18-44)	24 (17-35)	.007	26 (20-38)	23 (17-35)	.11
Graft surgery, *n* (%)	295 (9.7)	85 (29)	210 (71)		18 (8.0)	207 (92)	
In-hospital mortality, %	9.8	17	7.1	.02	44	7.7	<.001
Length of ICU stay, d, mean (CI)	6 (4-12)	5 (3-10)	6 (4-14)	.21	5 (3-7)	6 (4-11)	.16
Length of hospital stay, d, mean (CI)	24 (15-35)	26 (18-46)	23 (15-33)	.1	22 (6-28)	22 (15-37)	.20
Surgery for type A dissection, *n* (%)	1066 (35.1)	347 (33)	719 (67)		138 (15)	775 (85)	
In-hospital mortality, %	7.2	10	5.8	.01	17	4.9	<.001
Length of ICU stay, d, mean (CI)	5 (4-9)	6 (4-9)	5 (4-8)	.15	5 (4-8)	5 (4-8)	.91
Length of hospital stay, d, mean (CI)	24 (17-37)	26 (18-41)	23 (17-35)	.15	26 (18-36)	23 (17-35)	.19
Nonsurgery, *n* (%)	1820 (59.9)	273 (15)	1547 (85) (((85.0)		40 (4.3)	884 (96)	
In-hospital mortality, %	19	58	12	<.001	65	16	<.001
Length of ICU stay, d, mean (CI)	4 (2-6)	4 (2-6)	4 (2-6)	.89	6 (2-13)	4 (2-6)	.4
Length of hospital stay, d, mean (CI)	15 (7-22)	2 (1-18)	16 (10-22)	<.001	2 (1-25)	15 (9-22)	<.001

DIC, disseminated intravascular coagulation; ICU, intensive care unit. ISTH, International Society on Thrombosis and Haemostasis; JAAM-2, Japanese Association for Acute Medicine.

**Table 4:** Comparative analysis of DIC criteria using JAAM-2, ISTH, and modified ISTH scoring systems.

DIC subscore	JAAM-2 (*n* = 3037)		OR (95% CI)	ISTH (*n* = 1933)		OR (95% CI)	modified ISTH (*n* = 3037)		OR (95% CI)
	No. (%)	Mortality		No. (%)	Mortality		No. (%)	Mortality	
DIC criteria									
Negative	2400 (79)	9.6	1 [Reference]	1751 (91)	11	1 [Reference]	2557 (84)	11	1 [Reference]
Positive	637 (21)	31	4.16 (3.35-5.16)	182 (9.4)	27	3.14 (2.19-4.50)	480 (16)	32	3.94 (3.13-4.95)
Platelet subscore									
0	2430 (80)	11	1 [Reference]	1642 (85)	11	1 [Reference]	2660 (88)	12	1 [Reference]
1	403 (13)	20	1.91 (1.45-2.51)	247 (13)	16	1.53 (1.05-2.23)	317 (10)	23	2.17 (1.63-2.88)
2	NA	NA	NA	44 (2.3)	34	4.23 (2.22-8.04)	60 (2)	43	5.44 (3.22-9.18)
3	204 (6.7)	34	3.94 (2.87-5.41)	NA	NA	NA	NA	NA	NA
PT subscore									
0	2316 (76)	10	1 [Reference]	1591 (82)	10	1 [Reference]	2561 (84)	11	1 [Reference]
1	721 (24)	27	3.39 (2.74-4.20)	205 (11)	20	2.22 (1.52-3.24)	283 (9.3)	30	3.31 (2.49-4.39)
2	NA	NA	NA	137 (7.1)	23	2.60 (1.69-4.00)	193 (6.4)	28	3.04 (2.17-4.26)
FDP subscore									
0	1050 (35)	6	1 [Reference]	581 (30)	5.7	1 [Reference]	1050 (35)	6	1 [Reference]
1	727 (24)	10	1.75 (1.23-2.48)	NA	NA	NA	NA	NA	NA
2	NA	NA	NA	465 (24)	8.8	1.61 (1.00-2.58)	727 (24)	10	1.75 (1.23-2.48)
3	1260 (41)	23	4.61 (3.47-6.13)	887 (46)	18	3.63 (2.45-5.36)	1260 (41)	23	4.61 (3.47-6.13)
Fibrinogen subscore									
0	NA	NA	NA	1828 (95)	11	1 [Reference]	NA	NA	NA
1	NA	NA	NA	105 (5.4)	30	3.37 (2.16-5.26)	NA	NA	NA

DIC, disseminated intravascular coagulation; FDP, fibrin degradation product; ISTH, International Society on Thrombosis and Haemostasis; JAAM-2, Japanese Association for Acute Medicine; NA, not applicable; OR, odds ratio; PT, prothrombin time.

The severity of coagulopathy, characterized by thrombocytopenia, elevated PT-INR, prolonged aPTT, elevated D-dimer, and decreased fibrinogen levels, was associated with increased in-hospital mortality. This trend was consistently observed in both the surgery and nonsurgery groups (Table 5). Additionally, patients with D-dimer ≥40 (483/2860) were observed more frequently and were approximately 6 times more likely to die in hospital compared with the reference group (odds ratio, 5.96; 95% CI, 4.52-7.87; *P* < .001).

**Table 5:** Association between hospital mortality and severity of coagulopathy.

Severity of coagulopathy	All		Surgery		Nonsurgery	
	No./total (mortality)	OR (95% CI)	No./total (mortality)	OR (95% CI)	No./total (mortality)	OR (95% CI)
Platelet count, 10^3^/μL						
≥150	202/1975 (10)	1 [Reference]	34/648 (5.3)	1 [Reference]	168/1327 (13)	1 [Reference]
100-149	125/685 (18)	1.96 (1.54-2.50)	24/328 (7.3)	1.43 (0.83-2.45)	101/357 (28)	2.7 (2.05-3.61)
50-99	74/314 (24)	2.71 (2.01-3.65)	19/204 (9.3)	1.85 (1.03-3.33)	55/110 (50)	6.90 (4.59-10.4)
≤49	26/60 (43)	6.71 (3.95-11.4)	9/35 (26)	6.25 (2.72-14.4)	17/25 (68)	14.7 (6.23-34.5)
PT-INR						
0-1.19	150/1453 (10)	1 [Reference]	29/582 (5.0)	1 [Reference]	121/871 (14)	1 [Reference]
1.2-1.49	76/300 (25)	2.95 (2.16-4.02)	10/166 (6.0)	1.22 (0.58-2.56)	66/134 (49)	6.02 (4.08-8.88)
1.5-1.99	34/110 (31)	3.89 (2.51-6.02)	8/68 (12)	2.54 (1.11-5.81)	26/42 (62)	10.1 (5.25-19.3)
≥2.0	31/53 (39)	5.70 (3.21-10.2)	4/23 (17)	4.01 (1.28-12.6)	17/30 (57)	8.11 (3.84-17.1)
APTT, s						
0-39.9	288/2454 (12)	1 [Reference]	60/955 (6.3)	1 [Reference]	228/1499 (15)	1 [Reference]
40-59.9	57/206 (28)	2.88 (2.07-4.00)	6/97 (6.2)	0.98 (0.41-2.34)	51/109 (47)	4.90 (3.28-7.33)
≥60	35/68 (52)	7.98 (4.88-13.0)	5/29 (17)	3.11 (1.15-8.43)	30/39 (77)	18.6 (8.7-40.0)
D-dimer, μg/mL						
0-9.9	105/1550 (6.8)	1 [Reference]	19/488 (3.9)	1 [Reference]	86/1062 (8.1)	1 [Reference]
10-24.9	94/600 (16)	2.56 (1.90-3.44)	18/260 (6.9)	1.84 (0.95-3.56)	76/340 (22)	3.27 (2.33-4.58)
25-39.9	51/227 (23)	3.99 (2.76-5.77)	11/116 (9.5)	2.59 (1.19-5.60)	40/111 (36)	6.39 (4.09-9.99)
≥40	146/483 (30)	5.96 (4.52-7.87)	31/265 (12)	3.27 (1.81-5.91)	115/218 (53)	12.7 (8.97-17.9)
Fibrinogen, mg/dL						
≥200	146/1458 (10)	1 [Reference]	35/637 (5.5)	1 [Reference]	111/821 (14)	1 [Reference]
150-199	34/244 (14)	1.45 (0.97-2.17)	9/181 (5.0)	0.90 (0.42-1.91)	25/62 (40)	4.21 (2.45-7.24)
100-149	22/125 (18)	1.92 (1.17-3.14)	10/107 (9.4)	1.77 (0.85-3.70)	12/18 (67)	12.8 (4.71-34.8)
≤99	31/102 (30)	3.87 (2.46-6.09)	15/81 (19)	3.91 (2.03-7.53)	16/22 (73)	17.1 (6.54-44.5)

APTT, activated partial thromboplastin time; OR, odds ratio; PT-INR, prothrombin time-international normalized ratio.

**Figure 3:**
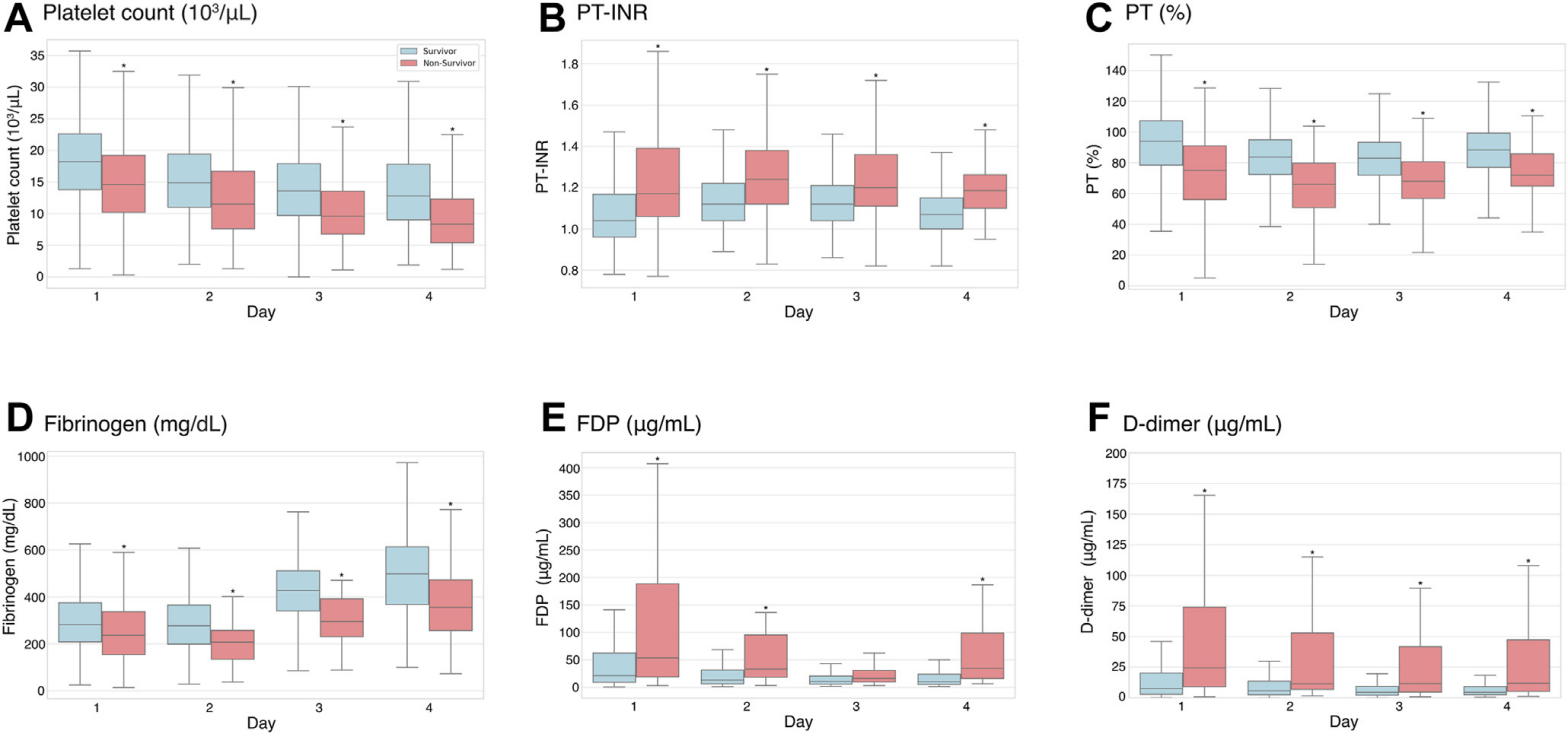
Box plot showing trends of (A) Platelet count, (B) PT-INR, (C) PT (%), (D) Fibrinogen, (E) FDP, and (F) D-dimer between survivor (blue boxes) and nonsurvivor (red boxes) from day 1 to day 4. ∗*P* < .001. vs survivor. FDP, fibrin degradation product; PT, prothrombin time; PT-INR, prothrombin time-international normalized ratio.

Figure 3 presents the trend of coagulation parameters from days 1 to 4, comparing survivors and nonsurvivors. Throughout these days, the nonsurvivor group consistently showed lower platelet counts, elevated PT-INR, reduced fibrinogen levels, and higher FDPs and D-dimer. While platelet counts leveled off from day 2 in the survivor group, they continuously declined in the nonsurvivor group over the observation period. Fibrinogen levels reached their lowest level on day 2, followed by a slower recovery in the nonsurvivor group than in the survivor group. Both FDPs and D-dimer were consistently higher in nonsurvivors than in survivors.

## 4 Discussion

This study investigated the epidemiology and impact of DIC in patients with acute aortic dissection. The clinical implications and transition of each coagulation/fibrinolysis marker were also evaluated. In summary, the prevalence of DIC was 21% with the JAAM-2 and 9.4% with the ISTH DIC criteria, and the presence of DIC was associated with increased in-hospital mortality. The results were consistent regardless of surgery, nonsurgery, or type of surgical intervention. As the JAAM-2 and ISTH DIC scores increased, the in-hospital mortality also increased. Additionally, patients with DIC required more blood transfusions, vasopressor use, mechanical ventilation, and renal replacement therapy. In-hospital mortality increased with the severity of coagulopathy, as characterized by various coagulation markers such as platelet count, PT-INR, aPTT, D-dimer, and fibrinogen. Considering that patients with DIC frequently require extensive interventions, a prompt and coordinated multidisciplinary treatment approach is essential to effectively manage these complex clinical scenarios.

Previous cohort studies have indicated an association between elevated D-dimer and reduced fibrinogen levels, and increased mortality in type A aortic dissection [17,18]. Additionally, consumption coagulopathy, marked by a reduction in clotting factor or fibrinogen levels during the perioperative period, has been reported [10]. However, these studies were limited to single-center settings, highlighting the need for more extensive verification. With >3000 patients included, this study is likely the most comprehensive to date, aiming to elucidate the clinical implications of DIC and various coagulation profiles and to offer new insights into the characteristics of coagulopathy in the acute phase of aortic dissection.

As expected, patients with acute aortic dissection exhibited a notable DIC prevalence, adversely affecting clinical outcomes. DIC associated with aortic dissection is characterized by decreased fibrinogen levels, markedly elevated FDP and D-dimer levels, and a high FDP/D-dimer ratio, unique to the fibrinolytic phenotype of DIC with enhanced fibrinolysis [19,20]. This may be biologically plausible because aortic aneurysms similarly show a fibrinolytic phenotype with annexin II expression in the aortic wall and a subsequent hemorrhagic tendency [21,22]. These results highlight the importance of managing enhanced fibrinolysis in the early phase of acute aortic dissection to control excessive bleeding effectively. Further research is essential to determine the optimal management strategies for DIC in patients with aortic dissection. Specifically, given the elevated FDP/D-dimer ratios observed in our cohort, studies investigating the potential role of antifibrinolytic agents, such as tranexamic acid, are warranted. Tranexamic acid has shown efficacy in managing coagulopathy with hyperfibrinolysis in other clinical settings, including trauma, obstetrics, and cardiovascular surgery [[23], [24], [25]]. However, its application in acute aortic dissection remains unexplored.

This study had some limitations. First, we excluded 2350 patients with missing data necessary to calculate the DIC score, which introduced a selection bias. To address this, we compared baseline characteristics and outcomes between the included and excluded cohorts. The excluded cohort had a higher proportion of patients who underwent surgery, required critical interventions (eg, vasopressor use, mechanical ventilation, and renal replacement therapy), and showed higher mortality (Supplementary Table S1) than the included cohort. Despite these differences, when we examined the relationship between DIC score subcomponents and mortality in both cohorts, we found a similar trend of increasing mortality odds with higher DIC subcomponents across both groups (Supplementary Table S2). Therefore, while selection bias remains a concern, the relationship between DIC development and mortality in acute aortic dissection did not appear to be significantly influenced by this bias in our study. Nevertheless, this study included a significantly larger number of patients than previous studies and has clinically important implications.

Second, although excessive bleeding is a critical complication of DIC in surgical patients, we could not examine the intraoperative information because it was not available in the current database. Third, the database did not contain information on whether the patients were taking anticoagulation or antiplatelet medications prior to admission. Furthermore, heparin was administered more frequently to patients in the DIC group, potentially affecting the coagulation status indicated by the DIC scores. Fourth, patients scheduled for surgery who died in the emergency room were included in the nonsurgical group, which may have contributed to the heterogeneity of patients in the nonsurgical group. Fifth, owing to the separate analysis of cohorts based on the availability of data for each criterion, a direct comparison between the JAAM-2 and ISTH criteria was not feasible. However, the analysis by each DIC subscore showed fibrinogen levels were below the threshold in only around 5% of cases using the ISTH score, which potentially reduced the sensitivity. On the other hand, low fibrinogen levels were strongly associated with mortality, which may contribute to an increase in the specificity of the ISTH score.

Sixth, the classification of DIC based solely on meeting scoring criteria presents significant diagnostic concern for acute aortic dissection. The diversion of blood into the false lumen mimics hemorrhagic loss, leading to reductions in circulating blood volume, platelets, and coagulation factors, which is difficult to distinguish from DIC. However, in trauma and postpartum hemorrhage, tissue damage, shock, and hypoxia contribute to the release of tissue plasminogen activator (t-PA), leading to a fibrinolytic phenotype of DIC [26,27]. In addition to the loss of platelets and coagulation factors due to bleeding, the increased t-PA activity further exacerbates coagulopathy and promotes subsequent bleeding. In the current study, DIC associated with aortic dissection is characterized by decreased fibrinogen levels, markedly elevated FDP and D-dimer levels, and a high FDP/D-dimer ratio, indicative of the fibrinolytic phenotype of DIC commonly observed in trauma and obstetric hemorrhage. Particularly in aortic aneurysm, Annexin II, a receptor for t-PA expressed in the aortic vessel wall, is implicated in potentially enhancing fibrinolysis [21,22]. Nevertheless, foundational research supporting the presence of DIC in acute aortic dissection remains insufficient, and further verification is necessary. Last, race/ethnicity data for participants were not collected in this study. However, given the ethnic homogeneity of Japan, where approximately 98% of the population is ethnically Japanese, it is reasonable to infer that the lack of this data likely does not significantly impact the overall results of the study.

## 5 Conclusion

This multicenter retrospective cohort study revealed that the identification of DIC, according to both the JAAM-2 and ISTH DIC criteria, was associated with higher in-hospital mortality in patients with acute aortic dissection. Further studies are warranted to determine the optimal treatment approach for patients with DIC and aortic dissection.

## References

[1] Hagan P.G., Nienaber C.A., Isselbacher E.M., Bruckman D., Karavite D.J., Russman P.L. (2000). The International Registry of Acute Aortic Dissection (IRAD): new insights into an old disease. JAMA.

[2] Mehta R.H., Suzuki T., Hagan P.G., Bossone E., Gilon D., Llovet A. (2002). Predicting death in patients with acute type a aortic dissection. Circulation.

[3] Ten Cate J.W., Timmers H., Becker A.E. (1975). Coagulopathy in ruptured or dissecting aortic aneurysms. Am J Med.

[4] Arima D., Suematsu Y., Yamada R., Matsumoto R., Kurahashi K., Nishi S. (2022). Relationship of acute type A aortic dissection and disseminated intravascular coagulation. J Vasc Surg.

[5] Gando S., Saitoh D., Ogura H., Mayumi T., Koseki K., Ikeda T. (2008). Natural history of disseminated intravascular coagulation diagnosed based on the newly established diagnostic criteria for critically ill patients: results of a multicenter, prospective survey. Crit Care Med.

[6] Sun Y., Wang J., Wu X., Xi C., Gai Y., Liu H. (2011). Validating the incidence of coagulopathy and disseminated intravascular coagulation in patients with traumatic brain injury--analysis of 242 cases. Br J Neurosurg.

[7] Sallah S., Wan J.Y., Nguyen N.P., Hanrahan L.R., Sigounas G. (2001). Disseminated intravascular coagulation in solid tumors: clinical and pathologic study. Thromb Haemost.

[8] Huang B., Tian L., Fan X., Zhu J., Liang Y., Yang Y. (2014). Low admission platelet counts predicts increased risk of in-hospital mortality in patients with type A acute aortic dissection. Int J Cardiol.

[9] Liu Y., Han L., Li J., Gong M., Zhang H., Guan X. (2017). Consumption coagulopathy in acute aortic dissection: principles of management. J Cardiothorac Surg.

[10] Guan X., Gong M., Wang X., Zhu J., Liu Y., Sun L. (2018). Low preoperative fibrinogen level is risk factor for neurological complications in acute aortic dissection. Medicine (Baltimore).

[11] Zhang Y., Li C., Shen M., Liu B., Zeng X., Shen T. (2017). Aortic aneurysm and chronic disseminated intravascular coagulation: a retrospective study of 235 patients. Front Med.

[12] Ogino H., Iida O., Akutsu K., Chiba Y., Hayashi H., Ishibashi-Ueda H. (2023). JCS/JSCVS/JATS/JSVS 2020 guideline on diagnosis and treatment of aortic aneurysm and aortic dissection. Circ J.

[13] Kaneko H., Itoh H., Kamon T., Fujiu K., Morita K., Michihata N. (2020). Association of cardiovascular health metrics with subsequent cardiovascular disease in young adults. J Am Coll Cardiol.

[14] Kaneko H., Yano Y., Itoh H., Morita K., Kiriyama H., Kamon T. (2021). Association of blood pressure classification using the 2017 American College of Cardiology/American Heart Association blood pressure guideline with risk of heart failure and atrial fibrillation. Circulation.

[15] Yamakawa K., Umemura Y., Mochizuki K., Matsuoka T., Wada T., Hayakawa M. (2024). Proposal and validation of a clinically relevant modification of the Japanese Association for Acute Medicine disseminated intravascular coagulation diagnostic criteria for sepsis. Thromb Haemost.

[16] Taylor F.B., Toh C.H., Hoots W.K., Wada H., Levi M. (2001). Scientific Subcommittee on Disseminated Intravascular Coagulation (DIC) of the International Society on Thrombosis and Haemostasis (ISTH). Towards definition, clinical and laboratory criteria, and a scoring system for disseminated intravascular coagulation. Thromb Haemost.

[17] Tian L., Fan X., Zhu J., Liang Y., Li J., Yang Y. (2014). Plasma D-dimer and in-hospital mortality in patients with Stanford type A acute aortic dissection. Blood Coagul Fibrinolysis.

[18] Liu J., Sun L.L., Wang J., Ji G. (2018). The relationship between fibrinogen and in-hospital mortality in patients with type A acute aortic dissection. Am J Emerg Med.

[19] Wada T., Gando S., Maekaw K., Katabami K., Sageshima H., Hayakawa M. (2017). Disseminated intravascular coagulation with increased fibrinolysis during the early phase of isolated traumatic brain injury. Crit Care.

[20] Asakura H. (2014). Classifying types of disseminated intravascular coagulation: clinical and animal models. J Intensive Care.

[21] Hayashi T., Morishita E., Ohtake H., Oda Y., Ohta K., Arahata M., Kadohira Y. (2008). Expression of annexin II in human atherosclerotic abdominal aortic aneurysms. Thromb Res.

[22] Hayashi T., Morishita E., Ohtake H., Oda Y., Asakura H., Nakao S. (2009). Expression of annexin II in experimental abdominal aortic aneurysms. Int J Hematol.

[23] Shakur H., Roberts I., Bautista R., Caballero J., Coats T., CRASH-2 trial collaborators (2010). Effects of tranexamic acid on death, vascular occlusive events, and blood transfusion in trauma patients with significant haemorrhage (CRASH-2): a randomised, placebo-controlled trial. Lancet.

[24] WOMAN Trial Collaborators (2017). Effect of early tranexamic acid administration on mortality, hysterectomy, and other morbidities in women with post-partum haemorrhage (WOMAN): an international, randomised, double-blind, placebo-controlled trial. Lancet.

[25] Myles P.S., Smith J.A., Forbes A., Silbert B., Jayarajah M., Painter T. (2017). Tranexamic acid in patients undergoing coronary-artery surgery. N Engl J Med.

[26] Kruithof E.K., Tran-Thang C., Gudinchet A., Hauert J., Nicoloso G., Genton C. (1987). Fibrinolysis in pregnancy: a study of plasminogen activator inhibitors. Blood.

[27] Cardenas J.C., Matijevic N., Baer L.A., Holcomb J.B., Cotton B.A., Wade C.E. (2014). Elevated tissue plasminogen activator and reduced plasminogen activator inhibitor promote hyperfibrinolysis in trauma patients. Shock.

