# Supplementary material for: Prevalence and clinical impact of disseminated intravascular coagulation in acute aortic dissection: a nationwide cohort study
Source: Res Pract Thromb Haemost. 2024 Dec 16;9(1):102656. doi: 10.1016/j.rpth.2024.102656 (PMC11774824; doi:10.1016/j.rpth.2024.102656)
Supplement: Supplementary Tables [file mmc1.docx]

**Supplementary Table1. Clinical characteristics of the study patients and the excluded patients**

|  | Included cohort | Excluded cohort | *p* |
| --- | --- | --- | --- |
| Number of patient (%) | 3037  2400 (79) | 2350  1751 (91) |  |
| Age, year | 73 (62-81)  72 (61-81)  <0.001 | 73 (62-82)  72 (60-80)  0.077 | 0.061 |
| Sex, male | 56  315 (49)  <0.001 | 52  969 (55)  0.247 | 0.004 |
| BMI | 23.0 (20.6-25.6)  23.2 (20.7-25.7)  0.075 | 22.7 (20.3-25.4)  23.1 (20.5-25.8)  0.744 | 0.012 |
|  |  |  |  |
| Comorbidity |  |  |  |
| Myocardial infarction | 124 (4.8) | 70 (3.0) | 0.037 |
| Heart failure | 1086 (36) | 886 (38) | 0.147 |
| Chronic lung diseases | 308 (10) | 212 (9.0) | 0.183 |
| Cerebral vascular diseases | 549 (18) | 398 (17) | 0.294 |
| Diabetes melitus | 366 (12) | 219 (9.3) | <0.001 |
| Chronic Kidney diseases | 248 (8.2) | 180 (7.7) | 0.531 |
|  |  |  |  |
| Surgery, n (%) | 1217 (40) | 1090 (46) | <0.001 |
| Open surgery, n (%) | 955 (32) | 1408 (40) | <0.001 |
| Graft surgery, n (%) | 295 (9.7) | 181 (7.7) | 0.01 |
| Surgery for type A, n (%) | 1066 (35) | 931 (40) | <0.001 |
| Surgery for type B, n (%) | 151 (5.0) | 159 (6.8) | 0.72 |
| Non surgery, n (%) | 1820 (60) | 1260 (54) | <0.001 |
|  |  |  |  |
| Coagulation test on admission |  |  |  |
| Platelet count, 10^3^/μL (n=3037) | 17,7 (13.2-22.2) | 16.8 (12.1-21.4) | <0.001 |
| PT, s (n=2890) | 12.4 (11.6-13.8) | 13.1 (11.9-15.0) | <0.001 |
| PT-INR (n=1943) | 1.06 (0.97-1.19) | 1.08 (0.98-1.25) | <0.001 |
| APTT, s (n=2728) | 29.7 (26.7-32.6) | 30.0 (27.1-33.2) | <0.001 |
| Fibrinogen, g/L (n=1933) | 279 (201-370) | 235 (159-320) | <0.001 |
| FDP, μg/mL (n=1538) | 23.3 (9.2-71.4) | 36.7 (11.5-76.8) | <0.001 |
| D-dimer, μg/mL (n=2860) | 8.6 (3.1-24.8) | 8.2 (3.2-20.5) | <0.001 |
| FDP/D-dimer ratio (n=1432) | 2.6 (2.2-3.2) | 3.1 (2.3-4.5) | <0.001 |
| Antithombin, % (n=626) | 88 (79-97) | 89 (77-98) | 0.358 |
| TAT, ng/mL (n=180) | 26.2 (12.2-50.3) | NA | NA |
| PIC, μg/mL (n=1) | 0.5 (0.5-0.5) | NA | NA |
|  |  |  |  |
| ICU admisssion, n (%) | 1672 (55) | 1288 (55) | 0.865 |
| Vasopressor use, n (%) | 1155 (38) | 995 (42) | <0.001 |
| Mechanical ventilation, n (%) | 1265 (42) | 1177 (50) | <0.001 |
| Renal replacement therapy | 180 (6.0) | 194 (8.3) | <0.001 |
| Red blood cell, U | 10 (6-16) | 10 (8-16) | 0.805 |
| Fresh frozen plazma, U | 10 (8-16) | 10 (6-14) | <0.001 |
| Platelets, U | 20 (10-20) | 20 (10-20) | <0.001 |
|  |  |  |  |
| In-hospital mortality, % | 14 | 17 | <0.001 |

BMI, body mass index; PT, prothrombin time; PT-INR, prothrombin time-international normalized ratio; APTT, activated partial thromboplastin time; FDP, fibrin degradation products; ICU, intensive care unit.

**Supplementary Table2. Mortality odds ratios according to the increase in JAAM-2 DIC subscores among included patients and excluded patients.**

|  | Included cohort | | Excluded cohort | |
| --- | --- | --- | --- | --- |
| JAAM-2 subscore | No. | OR (95% CI) | No. | OR (95% CI) |
| Platelet subscore |  |  |  |  |
| 0 | 2430 | 1 [Reference] | 1571 | 1 [Reference] |
| 1 | 403 | 1.91 (1.45-2.51) | 329 | 2.28 (1.69-3.06) |
| 3 | 204 | 3.94 (2.87-5.41) | 192 | 4.57 (3.29-6.35) |
| PT subscore |  |  |  |  |
| 0 | 2316 | 1 [Reference] | 1133 | 1 [Reference] |
| 1 | 721 | 3.39 (2.74-4.20) | 564 | 3.04 (2.31-4.01) |
| FDP subscore |  |  |  |  |
| 0 | 1050 | 1 [Reference] | 71 | 1 [Reference] |
| 1 | 727 | 1.75 (1.23-2.48) | 43 | 1.35 (0.34-5.34) |
| 3 | 1260 | 4.61 (3.47-6.13) | 80 | 5.01 (1.78-14) |

JAAM-2, Japanese Association for Acute Medicine; DIC, disseminated intravascular coagulation; OR, odds ratio; CI, confidence interval; PT, prothrombin time; FDP, fibrin degradation products.
